# Mr. Winterbottom, on Inoculation

**Published:** 1805-07-01

**Authors:** T. M. Winterbottom

**Affiliations:** South Shields


### To Dr. WILLAN.

SIR,

HAVING been extensively engaged in inoculating for the cow pock, I feel deeply interested for its success, and of course anxious to remove every ill-grounded report to its disadvantage The cow-pock inoculation has been favourably received in this town and neighbourhood, and continues to be very generally practised. Objections have occasionally been made to it by the ignorant and timid, which, however, have had no permanent effect upon the public mind.

About four months ago, whilst a bad kind of small pox was raging in the town, I was informed that a child, which had been vaccinated some months before, was affected with small pox: I therefore thought it my duty to enquire into the circumstances. Upon going to the house, I found a child, about seven years of age, labouring under the confluent small pox, and was informed, by the parents, that the youngest child, two years of age, had been inoculated twelve months before, for the cow pox, and, according to their account, the vesicle had passed through the regular stages. From this child matter was taken, on the seventh day, to inoculate the elder one, now ill, and, from the appearances which took place, the surgeon pronounced her to be secure from the small pox. About a fortnight before my visit, however, this child happening to go into a house where a person lay ill of the confluent small pox, immediately became sick, and returned to her mother, much alarmed, and complaining of the bad smell. Two days afterwards the small pox appeared, and were extremely severe. This case was certainly a very glaring instance of failure, and accordingly made much noise; but, upon inquiry, the business was soon unravelled. The punctured parts had inflamed considerably in the second child, immediately after inoculation, and had nearly subsided on the ninth day, when those of the child first inoculated only began began to inflame. Upon examination, a cicatrix was very visible on the arm of the younger child; but not a vestige was observable on that of the older one. During the whole progress of the disease, the younger child slept with its sister, and underwent the ordeal with impunity. It is therefore evident, from this account of the parents, that the older child had not received the infection of the vaccine pock.

Whilst this case was still talked of, two other children, that had been vaccinated some months before, were said to be affected with small pox, which induced me to visit them also. I found they had both experienced a slight indisposition, previous to the eruption of a number of pustules which contained a puriform fluid; but at my visit, on the sixth day of the eruption, the pustules had disappeared; not even a scab was visible, and the skin appeared only slightly discoloured with blotches. The arms of the children were visibly marked by the inoculation, which, by the account I received, had proceeded regularly. Several such instances of varicella have occurred in my own practice, without occasioning any uneasiness to the parents, because I had forewarned them of such an occurrence.

Since the above was written, four cases of small pox, after supposed vaccination, have occurred to Mr. G. a surgeon in this town. When informed of these unfortunate cases, I visited the patients, to be convinced of the truth of the report, and applied to the gentleman himself; but he had forgotten the circumstances respecting them, further than that he supposed they had passed regularly through the inoculation, as his other patients had done, and particularly his own child, which he had afterwards submitted to the test of variolous inoculation without effect. The following statement, with which he favoured me, is all the information I could procure concerning them:

"John Gait, son of John Gait, inoculated the 5th December, 1804; inflamed regularly, and pustules full, the 14th; left an indelible mark; took the natural pox the 3d March, 1805, confluent and bad kind; died the 14th.

"Robert Thompson, son of Thomas Thompson, inoculated the 5th March, 1804; inflammation and other symptoms regular; took the natural pox the 10th March, 1805, distinct and mild.

"Richard Hall, son of Richard Hall, inoculated the 17th December, 1804; symptoms regular; had four or five pustules on other parts, caused by scratching; took the natural pox the 24th February, 1805, full, but not confluent.

B 4 " —— Elder

"—— Elder was inoculated on the 20th December, 1804, and took the natural small pox in April; confluent and bad kind."

I call these cases unfortunate, because they did much mischief at the time: they created suspicion, and unsettled the mind of the public, though they did not, in the least, militate against the cow pock, as it had assuredly not been produced; they were instances of what has very injudiciously been called spurious cow pock; I have hinted this to Mr. G. and recommended him to reinoculate all the patients who were vaccinated at that time, as I am convinced they are in a precarious state. I have examined the arms of all the abovementioned children, except of that which died, and could not perceive the smallest appearance of depression or pit in the inoculated parts; but I must acknowledge that, in this respect, there is a great difference in my own patients: for in some the depression is considerable; while, in others, there is only a circular patch, of a deader white than the surrounding skin.

It appears astonishing that, at this advanced period of the practice, any mistake can occur, respecting the appearance of the vaccine pustule, when it has not been broken by scratching. Until the fourth or fifth day, sometimes a little later, it resembles a small pox pustule, has a small indentation in the centre, and a slight tinge of a roseate hue, which, as the vesicle approaches the size of a common pea, gradually changes into a dirty white, whilst the indentation becomes more deep. The circumference of the vesicle is bounded by a faint red circle, in some instances scarcely perceptible, which, about the 9th day, becomes diffused, and of a fiery red, having frequently a deep tinge or flush of red, shooting in a radiated manner from the centre to the circumference; but those who have noticed the seed of the common mallow, will instantly recognize the orbicular form, convex, and somewhat striated edges, and depressed centre of the cow pock vesicle. This criterion was, I believe, first noticed by the American Jenner, professor Waterhouse, in his instructive account of his labours in introducing the *kine pock* into America, with a perusal of which I was favoured by my friend, Dr. Willan.

During the last winter, the small pox and measles were epidemic in this town, and frequently appeared together in the same patient. The small pox was of a very malignant kind, and carried off a number of children. In consequence of this mortality, the applications for vaccine inoculation were numerous, and some of the children were of of course inoculated whilst under the influence of variolous contagion, or were exposed to it before the vaccine virus had affected the system; but in all these instances the cow pock vesicle proceeded regularly, except in appearing to be larger than usual, and having no areola. One child, inoculated with fluid vaccine matter, sickened four days afterwards, and, on the fifth, the confluent small pox appeared, which proved fatal on the fourteenth day. Matter taken from a vaccine pock of the ninth day, on this child's arm, perfectly formed and turgid, but without areola, was inserted into the arms of three children, who passed through the vaccination in the most regular manner. Another child, about five years of age, living in a house where a young man was brought, having a numerous eruption of discrete small pox, was immediately removed, and inoculated with vaccine fluid. The vesicles formed and proceeded as usual; on the eighth day after inoculation she sickened, and on the ninth day upwards of two hundred pustules appeared, which on the eighth day of eruption contained a purulent matter, and became rough at the apices. It seemed here as if the variolous eruption had received some check or modification from the inoculation, as the pustules did not exceed the size of a large pin's head, and had not an inflamed base.

The measles, though equally prevalent, were, in general, mild, and appeared in several children during the progress of inoculation, without producing any other effect than of preventing or retarding the appearance of the areola. From a child on the eighth day of vaccine inoculation, and the fourth of the eruption of measles, three children, who had not been affected with the latter disease, were inoculated, and passed through the vaccine without being affected with morbilous contagion.

A lady, between the fourth and fifth month of pregnancy, was seized with a violent febrile attack, followed by a copious eruption of measles, from which she recovered without inconvenience; but from that time the motion of the child ceased. About three months afterwards she miscarried; the child appeared to have been a long time dead: the cuticle had somewhat of a speckled appearance, but not so distinct as to enable me to determine that the child had been affected with measles.

I have never met with any eruptions succeeding vaccine inoculation; nor have I ever observed any troublesome sores, or other inconveniencies, arising from the punctured parts. I have already given my testimony in favour of vaccine inoculation, (Physical Journal, vol. 6 and 7) which succeeding succeeding experience has confirmed; but I must observe, that the mode of explaining some of the supposed failures of the cow pock, which have been lately published, by attributing them to local pustules having been formed, unaccompanied with constitutional affection, have rather surprized me. This doubt occurred to me before; and I must now declare, that if an *evident* affection of the system be essentially necessary, many of my patients are insecure; for, among several hundred patients, whom I have inoculated since the year 1801, a very large proportion suffered no *apparent* constitutional affection from the communicated disease, if indeed, from its mildness, it may be called a disease.

I am aware that an ingenious method has been proposed to determine this doubt, by repeated inoculations during the progress of the vaccine vesicle; but I am certain the patients whom I have to deal with would not submit to the plan; nor could any medical man find time to practise it, where his patients are widely spread. I am still, however, inclined to doubt of the necessity of strongly-marked constitutional affection, because, ever since I mentioned my notions respecting the supposed want of susceptibility of variolous contagion, in so large a proportion of subjects as is commonly imagined, I have inoculated a great number of adults with vaccine matter, who were positive they never had been affected with small pox, and yet I have not succeeded in exciting a local pustule. The result has been the same in every instance; the punctures inflamed for the first four or five days, became covered with a conical scab, and then disappeared.

It may appear too trifling to notice, but I am persuaded that much of our success, in inoculation, depends upon the mode of introducing the virus; and if punctures were used, instead of slight incisions or scratches, there would be fewer failures in communicating the cow-pock infection. By making two punctures in each arm, and using fluid matter where it can be obtained, I have succeeded in vaccinating patients who had been inoculated but a few weeks before, and were supposed to be unsusceptible of the infection.

Vaccination has no where been received with more eagerness, or practised with more zeal, than in Germany: besides their numerous journals, which teem with facts respecting this important practice, there is one, entitled "Schlesisch-Südpreussisches Archiv der, die Ausrottungspocken betreffenden Erfahuengen und Verhandlungen für Aerzte

Aerzte und Nichtarzte von Friese & Nowack, which is dedicated solely to the purpose of collecting whatever is important in this practice, from foreign as well as domestic sources. The following paper, by Dr. Udleray of Eutin (inserted in the Nordisches Archiv für Natur-und Arzney-wissenschaft. Herausg von Prof. Pfaff & Dr Scheel. See Kausch's excellent Journal, entitled Geist und Kritik der Medic. & Chirurg. Zectschriften Deutschlands) contains some interesting particulars respecting the "blue cow pock," (which, it seems, is popularly known in Germany, as well as in England) that may perhaps prove not unacceptable to your readers. Dr. H. was first informed by a farrier, that in Holstein whole herds of cows are frequently affected with this disorder, although the men who attend the horses are never employed in milking the cows. They are well acquainted with the clear blue pock described by Dr. Jenner, and distinguish it from the following kinds, which they consider as spurious.

1st. The black pock, which is broader than a "*Holstein schilling*," depressed in the middle, and filled with a thick pus. The servant girls, when infected, experience slight rigors, succeeded by increased heat, depression of strength, head-ach, and pain in the axillæ; the pustule degenerates into a painful spreading ulcer, difficult to heal.

2nd. The yellowish brown pock, not so broad as the former, but turgid, with a clear yellow fœtid ichor, and looks like an amber vesicle. The cows are more disordered by this than the black kind; the milk is diminished in quantity, and spoiled by milking, which occasions an almost insupportable smell. A healthy person, infected with this kind of pock, nearly lost a joint of his fore finger from the ulcer. These two kinds of cow pock were observed by Dr. Nisser of Seeberg, and are not mentioned by Dr. Jenner.

3d. The white cow pock, consisting of a large white vesicle, and producing inflammation and tumour upon the hands and arms, but no affection of the system; they are easily cured by domestic remedies. This kind is noticed by Jenner; and the following, the *wind pocken,* which produce ulceration on the teats of the cow, and the hands and arms of the milkers, are noticed by Pearson.

In a young woman, seventeen years of age, Dr. Hollway found a small vesicular eruption on the hands and fore-arms, attended with considerable heat and tumour; she had likewise, on both sides of her hands, a number of large flat blackish vesicles. The colour seemed to arise from the dark dark red basis of the vesicles shining through the semi-transparent cuticle. She had milked cows that had the black pocks upon their udders, and, a fortnight before Dr. H's visit, had a slight febrile attack, but felt no subsequent illness. She had passed through the small pox in 1791: hence he concludes this was not the genuine cow pock, as it excited both local and constitutional illness, because it is contrary to what happens when the cow pock is excited after the small pox

Dr. Hollway found, on further inquiry, that the protective power of the cow pock against small pox is well known in Holstein; and he relates several instances of adults who had experienced the cow pock during infancy, and who could not afterwards receive the small pox, either by inoculation or exposure to contagion. A woman assured him that, twenty-eight years before, in order to escape the small pox, she had endeavoured to get the cow pock by milking infected cows; but not succeeding, she made a slight scratch, and rubbed it with cow-pock matter; in consequence of which she sickened. Several years afterwards she was inoculated with small-pox matter, and also exposed to contagion, without effect. Two women also had the cow pock forty-five years before, and had ever since resisted the small pox; and another woman had, for thirty-five years, been protected in like manner. Dr. H. also mentions a farmer who had inoculated five of his children thirteen years before, with cow-pock matter; a practice which, it appears, had long prevailed in his family. In July of the same year (1802) Dr. H. visited a healthy girl, about eighteen, who had been affected with the cow pock, three weeks before; she had been sick about eight days, and had sometimes been confined to bed. She was now well; but had, upon her hands, some dry scabs of the cow pock. The pustules upon the teats of the cow were of a clear blue colour, before they burst. The crusts were removed from the girl's hands; and the thin fluid, upon the exposed parts, was absorbed on lint; and with this three children were inoculated next day, by means of small blisters. (This mode of inoculation, which was very generally practised on the continent, for communicating the small pox, cannot be too strongly reprobated in the present disease, as it destroys the characteristic appearance of the vesicle, upon which alone we can form our opinion of the patient's security). On the second day the inoculated parts discharged a little; a thin yellowish matter was observable on the fourth, which increased on the following days.

The

The night of the eighth was restless; and on the ninth a general indisposition took place, which continued until the twelfth. On the fifteenth all disorder had ceased; the inoculated spots formed considerable ulcers, which healed by the application of digestive ointment. Dr. H. observes that the inoculation succeeded with the crust or scab of the cow pock, as well as with that of the small pox. One of his patients, some weeks after inoculation, having scratched the inoculated part violently, was affected with tumour, and hardness of the upper part of the arm, which gradually spread over the fore-arm, but yielded at length to the application of lime water, and the internal use of the decoct. calc. antim. sulph. of Hoffman. A cow, which had ceased to give milk, was inoculated in the teat with vaccine virus. On the third day a redness appeared, which gradually increased; the teat was swelled on the eighth; on the eleventh the cow was hotter than usual, and the pulse beat sixty times in a minute. A hard ring, about five lines in diameter, appeared, in the midst of which a brown scab was observable on the thirteenth; the eyes were watery, and the pulse sixty-four. On the fourteenth the udder was hard; the ring of the vesicle was soft, more elevated, and had a blue tinge. On the fifteenth the scab appeared rather moist, and the next day it fell off.

*South Shields, May* 24, 1805. T. M. WINTERBOTTOM.

